# Diabetic foot care: knowledge and practice

**DOI:** 10.1186/s12902-020-0512-y

**Published:** 2020-03-20

**Authors:** Aydin Pourkazemi, Atefeh Ghanbari, Monireh Khojamli, Heydarali Balo, Hossein Hemmati, Zakiyeh Jafaryparvar, Behrang Motamed

**Affiliations:** 1 Razi Clinical Research Development unit, Guilan university of medical sciences, Rasht, Iran; 2 Social Determinants of Health Research center, nursing and midwifery school, Guilan University of medical sciences, Rasht, Iran; 3 Department of internal medicine , Razi Hospital ,School of Medicine, Guilan university of Medical Sciences, Rasht, Iran

**Keywords:** Diabetic foot, Diabetes mellitus, Knowledge, Practice, Foot ulcer

## Abstract

**Background:**

Diabetic foot ulcers (DFUs) are common problems in diabetes. One of the most important factors affecting the quality of diabetes care is knowledge and practice. The current study aimed at determining the knowledge and practice of patients with diabetes regarding the prevention and care of DFUs.

**Methods:**

The current analytical, cross sectional study was conducted in Guilan Province (north of Iran) on 375 patients registered in the medical records as type 2 diabetes mellitus. Demographic characteristics, knowledge, and practice of participants were recorded in a questionnaire during face-to-face interviews conducted by the researcher. Descriptive and inferential statistics were performed using SPSS version18.

**Results:**

The mean score of knowledge was 8.63 ± 2.5 out of 15, indicating that the majority of participants had a poor knowledge (84.8%). The mean practice score was 7.6 ± 2.5 out of 15, indicating that a half of them had poor performance (49.6%). There was a significant and direct correlation between knowledge and practice. Knowledge level, place of residence, marital status, and history of admission due to diabetic foot were predictors of practice score.

**Conclusions:**

According to the low level of knowledge and practice in patients with diabetes regarding the prevention and care of DFUs, and considering the significant relationship of some demographics of patients with knowledge and practice scores, a targeted educational program is needed to promote knowledge of patients with diabetes.

## What is already known about this subject?

- Diabetes accounted for 1.3 million deaths (2.4% of all death). The prevalence of diabetes varies among countries in Eastern Mediterranean Region (EMR).
- Prevalence of diabetes mellitus in Iran ranged 20 to 30% in different provinces with higher frequency among females from 1990 to 2013.
- Among people living with diabetes mellitus, 20% are at risk for foot ulceration as a result of neuropathy.
- Diabetic foot ulcers (DFUs) are one of most common diabetes complications with 0–4% prevalence.
- Good knowledge and practice regarding DFU reduces the risk of diabetic foot complications and ultimately amputation.

## What are the new findings?

- In the current study, 84.8% of the participants had poor knowledge and only 8.8% had good practice. There was a direct and significant correlation between knowledge and practice.

- The lowest knowledge scores belonged to the use of talcum powder or other powders and not using lotions between the toes.
- The strongest variables related to practice were knowledge, place of residence, marital status, and history of admission due to diabetic foot, indicating that these four variables were the predictors of practice score.

## How might this impact on clinical practice in the foreseeable future?

- Patients’ knowledge about foot ulcer prevention should be promoted based on guidelines both in community and hospitals.
- Adherence to guidelines prevents DFU; targeted interventions directed toward patients/health care providers can lead to reduced DFU complications.

## Background

Diabetes mellitus is a group of common metabolic disease characterized by hyperglycemia. Due to multiple and prolonged complications, diabetes affects almost all systems of the body [1]. Diabetes caused 1.3 million deaths (2.4% of all death) and 56 million disability adjusted life years (DALYs) in 2013. The diabetes DALY rate increased from 589.9 per 100,000 in 1990 to 883.5 per 100,000 populations in 2013. Total DALYs from diabetes increased by 148.6% during 1990–2013; population growth accounted for a 62.9% increase, and aging and increase in age-specific DALY rates accounted for 31.8 and 53.9%, respectively [2]. The prevalence of diabetes varies among countries in EMR. In Saudi Arabia, the prevalence of diabetes was reported 13.4% Saudis aged 15 years or older [3] and in Pakistan 12.1% for males and 9.8% for females aged ≥25 years [2]. A systematic review on the prevalence of type 2 diabetes in Iran showed a range of 3 to 20% in different provinces [4].

Of people living with diabetes, 20% are at high risk of foot ulceration as a result of neuropathy [5]. Diabetic foot ulcers (DFU_S_) comprise 12–15% of total estimated cost of diabetes in the developed countries, increasing to 40% in the developing countries [6]. DFUs are one of the most common diabetes complications with 4 to 10% prevalence in the affected population [7]. The overall incidence of DFU is 5.8–6.0% in some particular diabetic in the U. S, while it is 2.1–2.2% in smaller populations in Europe [8]. Treating foot ulcers can be expensive and it is evident that about 49–85% of all DFU_S_ can be prevented by raising awareness and taking proper measures [7]..

Among the complications of diabetes, DFU_S_ affects the patient’s quality of life in case of amputation. However, it is possible to prevent amputation using educational and care strategies [9]. Data show that 25% of patients with diabetes develop a foot ulcer in their lifetime and that the cost of treating a DFU_S_ is more than twice that of any other chronic ulcer [10]. Diabetic foot amputation remains an unpleasant impact on patients’ life more than other complications [11, 12]. Delays in referral of serious foot problems are of particular concern [5]. Ndosi et al., reported that 15.1% of patients died within the year of presentation, the ulcer had healed in 45.5%, but recurred in (9.6%). Participants with a single ulcer on their index foot had a higher incidence of healing than those with multiple ulcers (hazard ratio 1.90, 95% CI 1.18 to 3.06) [13].

Understanding the level of knowledge and practice in patients with diabetes is important in planning for the better control of diabetes and its complications. A study by Ahmad and Ahmad on 124 patients with diabetes in North India reported that 60.5 and 79.0% got lower scores in knowledge and practice toward diabetes, respectively [14]. Jackson IL et al., reported that 79.5% of patients with diabetes in Nigeria had more than 70% of overall knowledge about self-care [15]. The results of a study in Malaysia showed that the most patients (58%) had poor knowledge and 61.8% of them had poor practice of foot care [16].

Among diabetes complications, the foot ulcers are considered as the most preventable ones. Risk factors of DFU_S_ are correlated with poor practices and knowledge. Good knowledge and practice toward diabetic foot care reduces the risk of diabetic foot complications and ultimately amputation [7]. According to American Diabetes Association, annual assessments of knowledge, skills and behaviors are necessary for patients with diabetes [15].. The current study was conducted to assess patients’ knowledge and practice toward diabetic foot care. No similar study is conducted in Rasht City (the capital of Guilan Province, Northern Iran) thus far; therefore, the present study aimed at evaluating the level of practice and knowledge toward foot care in patients with type 2 diabetes mellitus. Health system can prevent DFU and amputation by applying a strategy to raise knowledge in patients.

## Methods

### Study design and subjects

The current analytical, cross sectional study was conducted at a clinic in Razi Hospital, affiliated to Guilan University of Medical Sciences, which is the only endocrine disease referral center across the province. Data were gathered from May to July 2017 and the subjects were selected by consecutive sampling. To Diagnostic and classify the patients, the American Diabetic Association, the diagnostic criteria were utilized [17]. Patients with diabetes receive care, education, treatment, and other services at this center. The center also delivers healthcare services to outpatients and inpatients, as well as routine training. The research project was approved by the Deputy of Research, Guilan University of Medical Sciences. Participation in the study was voluntarily and the subjects were informed about their right to withdraw from the study at any stage. The participant’s privacy was respected, and data were kept confidential and utilized for study purposes only. Participants were asked to read and sign an informed consent form. Inclusion criteria were: receiving the diagnosis of type 2 diabetes mellitus, age 18 years or above, taking anti-diabetic medications for at least 1 month prior to the study, having clinical records at the center, and willing to participate in the study. The exclusion criteria were: critically ill patients with diabetes, pregnant or newly diagnosed (less than 1 month) patients, receiving any other treatment or therapy, and having major psychiatric problems. A structured datasheet was used to collect demographic and clinical information of the patients using paper-based and digital records archives. Some information was also collected by a medical student through face-to-face interviews. A paper-based questionnaire was distributed among both outpatients and inpatients. Wagner DFU classification system was used to classify the patients based on ulcers. In this hospital, we assessed peripheral neuropathy, retinopathy and peripheral vascular disease (PVD), respectively by using monofilament testing, optometrist or ophthalmologist reports and the clinical diagnosis documented by the surgeon or, if available, images taken through arterial Doppler or angiography. Macro vascular disease was defined as any macro vascular complications other than PVD including prior myocardial infarction, angioplasty, coronary artery bypass grafting, ischemic heart disease, or stroke [18].

In the current study, having one or two more complications was considered a positive condition. The sample size was determined 375 considering 95% confidence interval with d = 0.05 and *P* = 0.58. A total of 375 out of 395 distributed questionnaires were completed and returned; the response rate was 94.4%.

### Measures

A three-section questionnaire was used in the current study. First section included demographic characteristics such as age, gender, and duration of diabetes mellitus, place of residence, occupation, and level of education, marital status, and body mass index. Second part consisted of 15 questions about knowledge scored based on nominal (yes/no/I don’t know) scale, and third part with 15 questions focusing on practice was scored based on “yes/no” scale. The questionnaire was used to measure the level of knowledge and practice of subjects toward diabetic foot care. Patients’ demographic data were collected to analyze factors associated with knowledge and practice toward diabetic foot care. Each correct answer was given 1 point; however, wrong answers or choosing “I don’t know” option was given 0 point. The total score for each part ranged 0 to 15. Good or poor level of knowledge was determined based on the 75% of the maximum score of the questionnaire; therefore, the scores higher than 11.25 were considered good and those lower than 11.25 were considered poor. Examples of the questions included “Do you care about your diabetes?”; “Do you wash your feet every day?”; “Do you check the water temperature before using it?” and “Do you dry your feet after washing?”

The questionnaire was translated into the Persian language. Following the translations conducted by an Iranian professor of English literature, a native bilingual English speaker translated it back into English. Content validity was determined by gathering the views of 15 medical and nursing professionals after reviewing the questionnaire. Content validity ratio (CVR) and content validity index (CVI) of the questionnaire were assessed. Mean scores of CVI and CVR were higher than 0.80. Cronbach’s α coefficients were computed to evaluate reliability of knowledge and practice, which were 0.80 and 0.85, respectively.

### Statistical analysis

After collecting data, descriptive statistics (frequency, mean, and standard deviation) were employed to summarize patients’ socio-demographic data and Chi-square test to investigate association between predictors (factors) and knowledge and practice level. In order to assess the differences between groups, the Wilcoxon, Mann-Whitney, and Kruskal-Willis tests were used for continuous variables. Factors related to knowledge and practice was estimated by multiple regressions. In this research, wrong answers and “I don’t know” merged as poor awareness. In order to assess the relationship between individual variables with knowledge and practice, we had to integrate these two items in order to have a better analysis. Variables with a *P*-value of < 0.1 were included in the multi-variate models. *P*-value < 0.05 was considered as the level of significance. All analyses were performed using SPSS version 18.

## Results

The mean (± SD) age of the 375 participants was 55.4 (±12.9) years, and 56.4% were female. Majority of patients had diabetes for less than 10 years (54.1%), were female (56.5%), urban residents (62.1%), illiterate or had elementary education (73.1%), did not have normal BMI (69.8%), and (10.6%) patients had 2 and more complications (Table 1). In terms of knowledge, only 57 participants (15.2%) had good knowledge, most of them (84.8%) had poor knowledge, and the mean score of patients’ knowledge was 8.63 ± 2.65. The highest percentage of correct answers was found with the knowledge about “The need for meeting or consulting a physician, if there were signs of wounding” (88.8%), followed by “Not walking without shoes” (83.5%) and “Washing and changing socks” (9.81%). The lowest knowledge was about “The use of talcum powder or other powders between the toes” (3.5%), followed by “Not using lotion between the toes” (22.24%), and “The proper method of trimming the toenails” (23.2%).

**Table 1:** Demographic Data of Participants

Characteristics	Gender		Total (*N* = 375)
	Female (*n* = 212)	Male (*n* = 163)	
Age, yrs. (mean ± SD)	54.62 ± 12.48	56.40 ± 13.31	55.4 ± 12.9
Education			
Illiterate	80 (37.7)	17 (10.4)	97 (25.9)
Read and write	5 (2.3)	15 (9.2)	20 (5.3)
Primary	85 (40)	72 (44.7)	157 (41.9)
Diploma	29 (13.6)	33 (20.2)	62 (16.5)
University	16 (7.5)	23 (14.1)	39 (10.4)
Marital status			
Single	200 (94.3)	143 (87.7)	30 (8)
Married	10 (4.7)	20 (8)	343 (91.4)
Divorced	1 (0.5)	0 (0)	1 (0.2)
Widowed	1 (0.5)	0 (0)	1 (0.2)
Occupation			
Civil servant	9 (4.2)	16 (9.8)	25 (6.7)
Self-employed/business	5 (2.3)	37 (22.6)	42 (11.2)
House wife	166 (78.3)	0 (0)	166 (44.3)
Student	7 (3.3)	6 (3.6)	13 (3.5)
Farmer	17 (8)	33 (20.2)	50 (13.3)
Retired	8 (3.7)	71 (43.5)	79 (21.1)
Place of residence			
Urban	135 (63.7)	98 (60.1)	233 (62.1)
Rural	77 (36.3)	65 (39.9)	142 (37.9)
Duration of diabetes (yrs.)			
< 10	116 (54.7)	87 (53.4)	203 (54.1)
> =10	96 (45.3)	76 (46.6)	172 (45.8)
Diabetic foot ulcer			
Yes	36 (17)	51 (31.3)	87 (23.2)
No	176 (83)	112 (68.7)	288 (76.8)
History of amputation			
Yes	23 (10.8)	37 (22.7)	60 (16)
No	189 (89.2)	126 (77.3)	315 (84)
History of hospitalization			
Yes	31 (14.6)	45 (27.6)	76 (20.2)
No	181 (85.4)	118 (72.4)	299 (79.7)
Body mass index (kg/m^2^)			
Underweight	3 (1.4)	4 (2.5)	7 (1.9)
Healthy weight	51 (22.1)	62 (38)	113 (30.1)
Overweight	92 (43.5)	80 (49.1)	172 (45.9)
Obesity	66 (31.1)	17 (10.4)	83 (22.1)
Complications(> = 2)			
Yes	10 (4.71)	30 (18.4)	40 (10.6)
No	202 (95.2)	133 (81.5)	335 (89.3)
Family history of diabetes			
Yes	20 (14.8)	40 (24.5)	60 (16)
No	192 (90.5)	123 (75.4)	315 (84)
Current smoker			
Yes	15 (7.07)	89 (54.6)	104 (27.7)
No	197 (92.9)	74 (45.3)	271 (72.2)

In terms of practice, only 33 patients (8.8%) had a good practice; most of them (91.2%) had a poor practice (Table 2), and the mean score of patients’ practice was 7.6 (± 2.5). The participants reported their best practice toward “Importance of diabetes control” (80.5%), followed by “Meeting or consulting a physician, in case of signs of DFU” (79.2%). The poorest practice was toward “The use of talcum powder between the toes” (2.7%), followed by “Proper method of trimming the toenails” (25.9%), and “Keeping the foot skin soft” (30.9%).

**Table 2:** Distribution of Patients According to Knowledge and Practice

Variable	Good Score			Poor Score		
	Female	Male	Total	Female	Male	Total
Knowledge	39 (18.4)	18 (11)	57 (15.2)	173 (81.8)	145 (89)	318 (84.8)
Practice	24 (11.4)	9 (5.5)	33 (8.8)	188 (88.7)	154 (94.5)	342 (91.2)

**Fig. 1:**
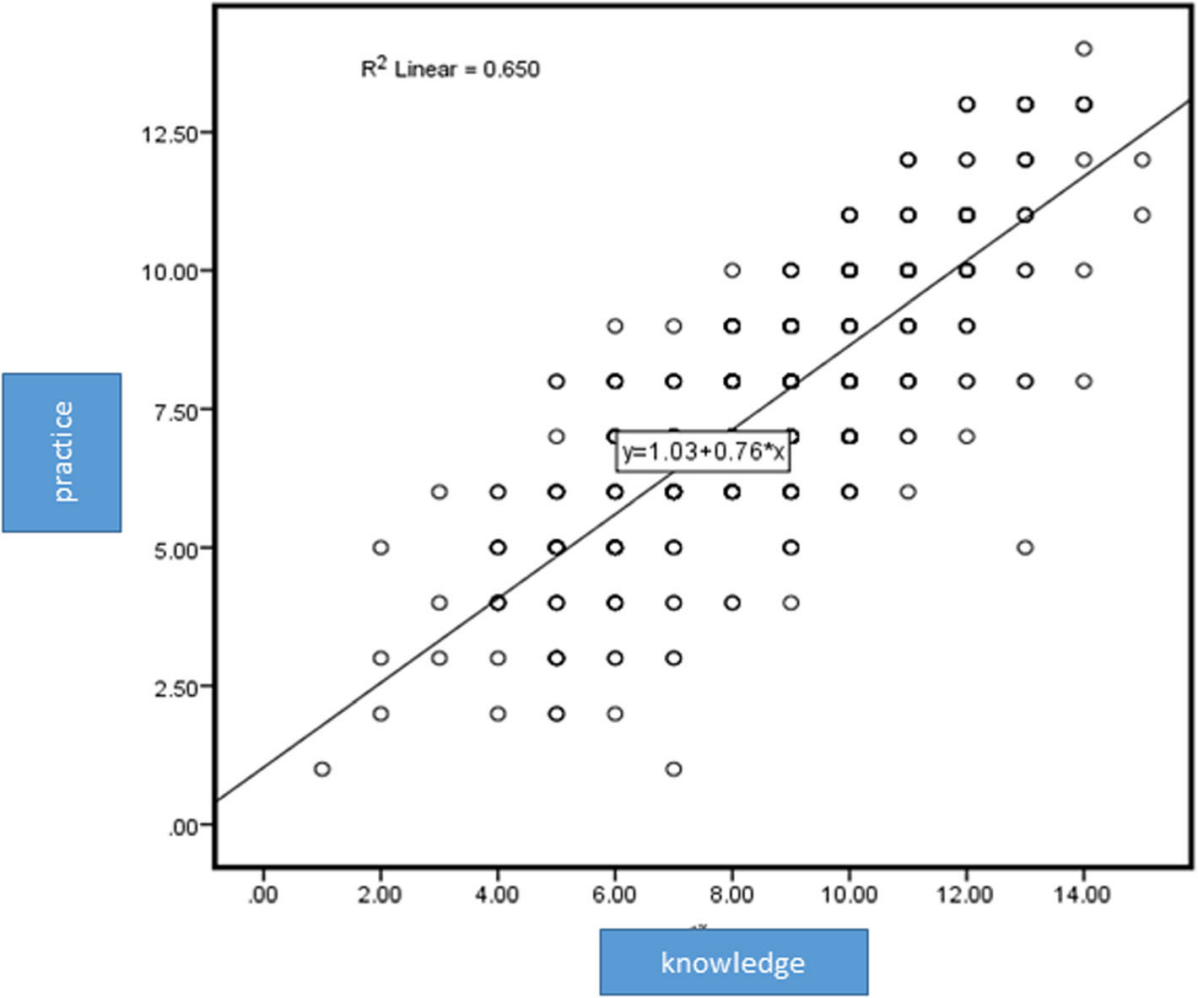
Correlation Between Khowledge and Practice

There was a direct and significant correlation between knowledge and practice (*P* < 0.0001, r < 0.8) (Fig. 1). There was a significant relationship between knowledge score and gender, duration of diabetes, occupation, level of education, place of residence, having DFU, hospital stay history, and amputation history.

The study results showed that patients with more than 10 years history of diabetes, history of DFU, history of hospital stay or experience of lower limb amputation due to DFU, female gender, and the ones with complications had higher knowledge (*P* < 0.05).

There was a significant correlation between practice score and gender, duration of diabetes, occupation, level of education, and place of residence (P < 0.05) (Table 3).

**Table 3:** The Relationship of Individual, Social, and Disease-dependent Variables With Knowledge and Practice

Variable	Knowledge	Practice
Age, yrs.	*P* = 0.72	*P* = 0.71
Gender	*P* = 0.0001	P = 0.0001
Duration of diabetes (yrs.)	*P* = 0.005	*P* = 0.016
Place of residence	*P* = 0.003	P = 0.0001
Occupation	P = 0.0001	P = 0.0001
Level of education	P = 0.0001	P = 0.0001
Marital status	*P* = 0.65	*P* = 0.14
Body mass index, kg/m^2^	*P* = 0.88	*P* = 0.33
Diabetic foot ulcer	*P* = 0.04	P = 0.5
History of hospital stay	*P* = 0.007	P = 0.14
History of amputation	P = 0.02	P = 0.5
Family history of diabetes	P = 0.5	P = 0.65
Complications	P = 0.02	P = 0.14
Current smoker	P = 0.14	P = 0.5

Also, based on multiple regression, the strongest variables related to practice were knowledge score (*P* < 0.0001), place of residence (*P* < 0.03), marital status (*P* = 0.008), and DFU (*P* = 0.02), indicating that these four variables were the predictors of foot care practices in the current study (Table 4).

**Table 4:** Multiple Regression of Predictor Factors of Practice Score

Variable	Unstandardized Coefficients(B)	Standard Error	Standardized Coefficients(β)	T	SIG	95%CI interval	
						Lower Limit	Upper Limit
Knowledge	0.75	0.03	0.79	10.74	0.0001	0.69	0.80
Place of residence	- 0.59	0.15	- 0.11	2.16	0.0001	−0.90	−0.29
(rural to urban)							
Marital status	−0.20	0.27	−0.08	4.30	0.008	−1.29	−0.19
(single to married)							
Diabetic foot ulcer	0.43	0.18	0.06	2.8	0.02	0.06	0.80

## Discussion

In the current study, majority of patients with diabetes had lower levels of education. Studies report that level of knowledge depends on the level of education [14, 19]. Understanding this variable is highly important in designing strategies to prevent diabetes.

In the current study, most patients had lower scores of knowledge and practice toward foot care, and the mean practice score was lower than the mean knowledge score, which was similar to the findings of Muhammad-Lutfi’s and Kim’s studies [16, 20]. A study conducted on patients with diabetes in Western Nepal reported poor KAP (knowledge, attitude and practices) score; they indicated that the plausible factors could be lack of knowledge, lack of information, and literacy level of the studied population [21]. Another study on young Saudi females with diabetes also reported poor KAP scores [19]. Some studies reported that patients with diabetes had good level of knowledge about diabetes [7, 16, 22, 23]. The differences in knowledge about foot care among patients with diabetes across the studies could be due to different trainings on diabetes care provided by the health care professionals in different settings [23] and also the literacy level of the studied subjects.

Several studies reported poor foot care practices among patients with diabetes. Kheir et al., reported poor practices toward regular inspection of feet among patients in Qatar [24]. Hamidah et al., from Malaysia observed that 28.4% of patients newly diagnosed with diabetes practiced good habits towards foot care [25]. Desalu et al., from Nigeria observed that only 10.2% of patients with diabetes had good foot care practices [26]. It was difficult to compare the results of the current study with those of other studies since the nature of the study populations and the applied measurements were different.

In the current study, there was a direct and significant correlation between knowledge and practice scores; therefore, with an increase in the knowledge score, the practice score also increased. Other studies also showed that patients who receive trainings on foot care checked their feet regularly [20]. Patients who are advised to take care of their feet and the ones whose feet are regularly checked by physicians have better practices toward foot care [27].

In the current study, the lowest knowledge scores were regarding the application of talcum powder or other powders and not using lotions between the toes, and the proper way of trimming the toenails; while the lowest practice scores were related to the application of talcum powder between the toes, the proper way of trimming the toenails; keeping the foot skin soft, and avoid dryness.

It should also be noted that due to wet climate in the North of Iran, use of lotion between the toes is not common. Nevertheless, it also needs training. Patients with diabetes need to keep between their toes dry using talcum powder and avoid the application of lotion since it is important as a hygienic measure for feet in preventing fungal infection [28]. Patients should also use skin moisturizers daily to keep the skin of their feet soft and should trim their toenails straight across (not rounded) to prevent damage to their toes [29].

In the current study, gender, duration of disease, occupation, place of residence, level of education, having DFU, and a history of hospitalization, amputation, and complication had significant relationships with knowledge. Also, gender, duration of disease, place of residence, occupation, and level of education had significant relationships with practice. It was found that knowledge level was higher in females, patients with a diabetes history of more than 10 years, and the ones underwent amputation due to DFU compared to the others; in addition, females, patients with a diabetes history of more than 10 years, and urban residents had better performance. The current study results showed that males were usually reluctant to disclose their health problems and seek professional care. Also, males presented greater deficit in self-care compared to females [30].

In the study by Muhammad-Lotfi, age, gender, level of education, and duration of diabetes had no significant relationship with knowledge and practice. This finding was in agreement with that of the current study [16], but another study indicated a significant relationship between the level of education and knowledge [31].

People with higher education are expected to be more likely to read and receive information about their illness and foot care and understand the information provided by medical staff in health care settings.

But in the current study, there was no significant relationship between the level of education and knowledge or practice, which could be due to the poor and inadequate resources of information about diabetes at the community level, since both educated and uneducated groups had inadequate information. It may also be due to the fact that in spite of possessing knowledge, due to the lack of time, heavy work load, and lack of adequate insurance coverage, patients could not take good care of their feet in practice, which requires more studies to root out the causes.

Nevertheless, the attitude of patients toward self-care in addition to sufficient knowledge was not studied in the current study. As observed in the present study, patients with a history of DFU or hospital stay, and even amputation and complication had higher knowledge level. It could be due to the fact that while completing the questionnaire, the current knowledge level of the subjects was questioned, which indicated that training medical centers can raise the level of knowledge in patients with DFU. In many Iranian state hospitals, diabetic training programs are not well organized, and the existing programs are weak. It is believed that knowledge about diabetes in the general population as well as patients with diabetes in Iran is not enough and there is a dire need for a good program for diabetes [32].

The collected data indicated that patients with diabetes had poor practice and knowledge about foot care. This is basically due to lack of proper communication between patients and medical team and inadequate education. Based on nurses’ opinion, recommendations and guidelines play an effective role in prevention, treatment, and reduction of complication among patients with DFU. Therefore, adaptation, implementation, and evaluation of the educational programs were recommended [33].

Thus, patients should be trained for foot ulcer prevention based on clinical practice guidelines for diabetes mellitus both in the community and hospitals. The results of the current study encouraged a positive outlook: A diabetes educator should give necessary advices to patients during every visit, in order to improve their perception about disease, diet, and lifestyle changes and help them control their glycemic level and overcome the complications of diabetes.

According to the principle of “prevention is better than cure” and considering the predictive factors in the current study including poor knowledge, urban residency, being single, and lack of DFU, more attention should be paid to patients possessing risk factors .

Knowledge and practice toward foot care were poor in most patients with diabetes. There was a significant relationship between some demographic characteristics of patients and knowledge and practice toward foot care. The level of knowledge, place of residence, marital status, and history of hospital stay due to DFU were the predictors of practice in patients with diabetes.

The strength of the current study was that it was the first, study to discuss this important issue in Guilan Province. The study also had some limitations; first, since the work had a cross sectional design, the direction of relationships and causal relationships cannot be determined. Second, the result of the study should be interpreted with caution, since they were obtained from a single center; a clinic-based study. Hospital-based studies cannot provide a true picture of knowledge and practice in the community. The current study sample did not represent the whole Iranian population consisting of several ethnicities. In this research, responses of the wrong answers and “I don’t know” have been grouped together, in order to achieve better analysis. Perhaps with increasing sample size, we could solve this problem in future studies.

## Conclusions

Adequate knowledge and good practices are important to effectively control diabetes mellitus. Patients require continuous support of family members and community in order to modify their lifestyle and behaviors and make sustainable changes in order to better control their diabetes disease. Also, education about diabetes mellitus and its risk factors should be provided through mass media in order to effectively control it in the community.

## Data Availability

The datasets used and /or analyzed during the current study are available from the corresponding author on reasonable request.

## Abbreviations

CVI: Content validity index
CVR: Content validity ratio
DFU: Diabetic foot ulcers
EMR: Eastern Mediterranean Region
WHO: World health organization
